# Retroperitoneal Malignant Peripheral Nerve Sheath Tumor Replacing an Absent Kidney in a Child

**DOI:** 10.1155/2013/627472

**Published:** 2013-12-10

**Authors:** Samin Alavi, M. T. Arzanian, Yalda Nilipour

**Affiliations:** ^1^Pediatric Congenital Hematologic Disorders Research Center, Shahid Beheshti University of Medical Sciences, Tehran 15468-15514, Iran; ^2^Mofid Children's Hospital, Dr. Shariati Avenue, Tehran, Iran; ^3^Pediatric Pathology Research Center, Mofid Children's Hospital, Shahid Beheshti University of Medical Sciences, Tehran 15468-15514, Iran

## Abstract

Malignant peripheral nerve sheath tumors (MPNSTs) are nonrhabdomyosarcoma soft tissue sarcomas with rare occurrence in children specially in the retroperitoneum. We describe a young child who presented with an abdominal mass. Both ultrasound and computed tomography revealed a large right-sided abdominal mass in the anatomic place of right kidney, while no kidney or ureter was observed at that side. He underwent surgical resection of the tumor with a primary impression of Wilms tumor. To the authors' knowledge, this is the first case of retroperitoneal malignant peripheral nerve sheath tumor and absent kidney. This case suggests the very rare probability of association of MPNSTs in children with genitourinary tract anomalies such as renal agenesis.

## 1. Introduction 

Soft tissue sarcomas (STSs) account for less than 1% of all cancer diagnoses in the general population. They are more common in children, representing approximately 7% of all cancers in patients younger than 20 years. Rhabdomyosarcomas comprise approximately half of the pediatric STSs. The remaining known as nonrhabdomyosarcoma soft tissue sarcomas (NRSTSs) are a heterogeneous group of neoplasms [1]. Malignant peripheral nerve sheath tumors (MPNST) are defined as any malignant tumor deriving from or differentiating into cells of the peripheral nerve sheaths with nonspecific symptoms and a high risk of local recurrence and distant metastasis [2]. MPNST is particularly rare with an incidence of 0.001% in the general population [1, 2]. It is a very rare spindle cell sarcoma in children accounting for approximately 5–10% of nonrhabdomyosarcoma soft tissue sarcomas [2, 3]. Herein, we report the first case of retroperitoneal MPNST that was associated with absent right kidney and compare our findings with the previous reports in the literature.

## 2. Case Presentation 

**Figure 1:**
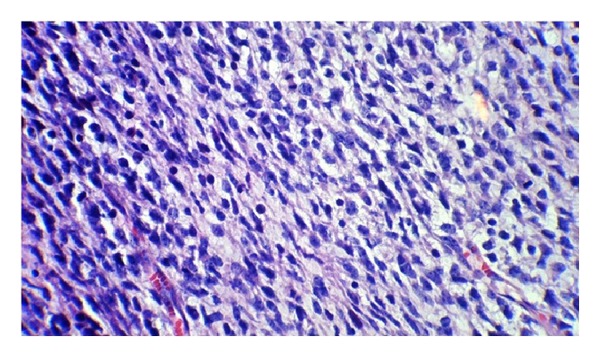
Soft tissue sarcoma with numerous mitotic figures (H&E×400).

**Figure 2:**
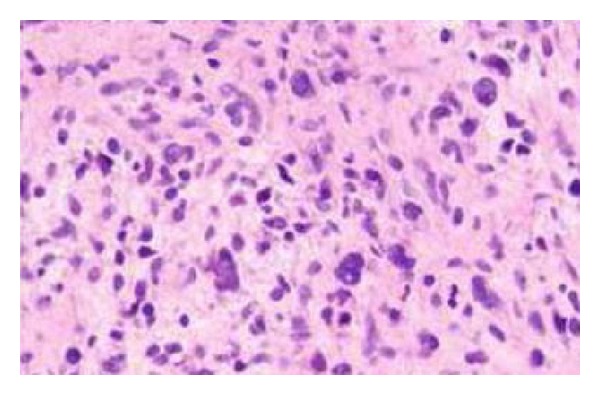
Nuclear hyperchromasia, enlargement, and crowding in MPNST (×400).

A 12-year-old boy was referred to pediatric clinic with abdominal pain lasting for one month. He was also complaining from nausea and vomiting during the week prior to his referral. On physical examination a huge abdominal mass mainly in the right side of the abdomen was detected. Abdominal ultrasonography showed a large solid/cystic, fluid filled mass measuring 17 × 17 cm in upper abdomen assumed to be originated from right kidney. In computed tomography with contrast the right kidney was not visualized, but a huge heterogeneous cystic, septated mass measuring 12 × 16 cm in the anatomic area of right kidney, extending upwardly to the diaphragm and liver and anteriorly to the pancreas, was noticed. Absence of right kidney was consistent with its agenesis. The patient was scheduled for laparotomy through which a huge tumoral mass with attachments to the diaphragm was removed. However, neither the right kidney nor the ureter was found during the operation. Macroscopic examination of the mass showed lobulated fragments of spherical tissues weighing about 630 gr with gelatinous and solid, hemorrhagic areas. Microscopically, a neoplasm consisting of serpentine wavy spindle cells with low cellularity in a myxoid stroma transforming to the hypercellular pleomorphic areas composed of atypical round to spindle cells with hyperchromatic, pleomorphic nuclei showing about more than 10 mitotic figures in 10 HPF was observed. The findings were suggestive of neurofibroma transforming to malignant peripheral nerve sheath tumor (MPNST), while no evidence of renal tissues was observed and margins of tissues were free of any tumoral cells (Figures 1 and 2).

**Figure 3:**
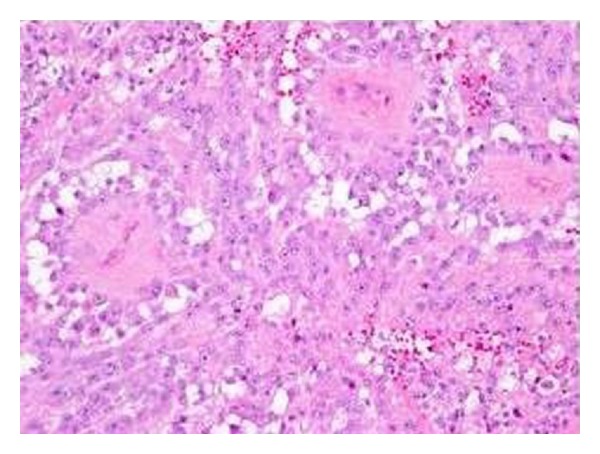
IHC showing cytoplasmic S-100 positivity in MPNST.

**Figure 4:**
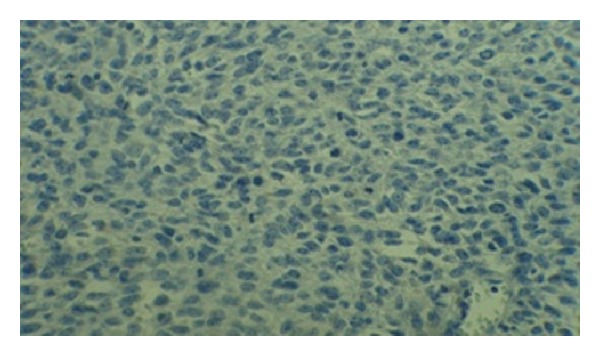
CD57 positivity with mitotic figure in tumoral cells.

Immunohistochemical markers including smooth muscle actin, cytokeratin, CD34, and c-Kit were all negative except for protein S-100 and CD-57 which were positive; the markers all related to nerves and nerve sheath, respectively (Figures 3 and 4). Chest computed tomography, bone marrow aspiration, and bone scan as part of systemic evaluation of the patient yielded normal findings. The patient received 8 courses of chemotherapy with carboplatin, doxorubicin, and ifosfamide for MPNST. Imaging study of the abdomen was normal during the treatment. He is in complete remission with no evidence of tumor recurrence after 3 years of follow-up.

## 3. Discussion

Malignant peripheral nerve sheath tumor (MPNST) replaces the former terminology of malignant schwannoma, malignant neurilemmoma, malignant neurofibroma, and malignant neurofibrosarcoma. They are highly malignant and locally invasive sarcomas and do not arise from malignant degeneration of schwannoma [3]; instead, they arise de novo or from transformation of a plexiform neurofibroma, so they have a significant association with von Recklinghausen neurofibromatosis. The lesions are poorly defined tumor masses with frequent infiltration along the axis of the parent nerve as well as invasion of adjacent soft tissues [2]. The etiology is unknown but there is a higher incidence in patients with a history of radiation exposure and von Recklinghausen neurofibromatosis [3]. In the present case, a neurofibromatous lesion transforming to malignant peripheral nerve sheath tumor (MPNST) was evident on pathology, although clinically our case lacked any evidence of von Recklinghausen neurofibromatosis and this discrepancy between clinical manifestation and pathology was a very interesting aspect of our case.

It is critical to distinguish schwannoma of atypical or cellular type from malignant peripheral nerve sheath tumor. Schwannoma (neurilemmoma) rarely arises from the kidney [4], is usually located in the renal hilum or pelvis, and clinically behaves in a benign fashion. Schwannoma consists of a nearly pure population of cells showing schwannian differentiation and arising eccentrically from the surface of their nerve of origin. The main nerves of the kidney consist of sympathetic and parasympathetic fibers that accompany the renal artery entering the renal hilum. This could explain the more frequent location of schwannomas at the renal hilum [4]. However, since the kidney was absent in our patient the tumor was assumed to be originated from somewhere else different from the kidney.

MPNSTs encompass a wide extent of biological behavior ranging from low to high grade malignancy and a variety of clinical manifestations; hence, it is not surprising to find that the genetic and molecular findings in these tumors reflect a heterogeneous mixture of various aspects of MPNSTs [5]. MPNSTs make up 5 to 10% of nonrhabdomyosarcoma soft tissue sarcomas in children and originate from peripheral nerves' sheath such as schwann cells, perineural cells, or fibroblasts [3]. A recent review of 120 MPNSTs during a 71-year period at Mayoclinic demonstrated an incidence of 4.6% in patients with von Recklinghausen's disease compared to the 0.001% incidence in general population [6]. MPNSTs usually present as an enlarging palpable mass and pain is a variable complaint [1, 2, 6]. The present case was a 12-year-old boy with abdominal pain, nausea, vomiting, and huge abdominal mass. There are several reports in the literature demonstrating the nerve sheath tumors can grow to giant sizes. Kuznetsov et al. reported a schwannoma of 2.89 kg [7]. During tumor formation, some Schwann cells degenerate, driving other schwann cells into the cycle. This recruits inflammatory cells into the nerve, ultimately leading to the formation of large tumors [8]. Our patient had an abdominal mass weighing about 630 gr.

CT-scan and MRI are more accurate than ultrasonography and can improve visualization of such abdominal masses. However, magnetic resonance imaging is the imaging modality of choice; a preoperative diagnosis is extremely difficult [9].

MPNSTs are most likely to metastasize to the lungs, followed by the bone and finally the pleura [3]. For this reason, a computed tomography of the chest is the preferred imaging study to screen for distant disease. A bone scan should also be obtained to help identify metastatic bone disease.

The clinical diagnosis of MPNST is difficult because of its rarity. Criteria for the diagnosis of MPNSTs are a controversial subject and include demonstration of origin of the tumor from a nerve, presence of Schwann cells, presence of neurofibroma transforming to a malignant sarcoma, nuclear palisading on microscopy and immunohistochemical findings of S-100 protein [10]. With regard to demonstration of nerve of origin at surgery, Dasgupta et al. observed that this is often not possible in view of the fact that they arise from small and insignificant nerves [11]. Nambisan et al. could not demonstrate the nerve origin of MPNST in about 61% of their cases [12], maybe due to the same problem.

Immunohistochemistry and electron microscopic studies have been gradually replacing histopathology as confirmatory diagnostic methods because of a wide variety of histological patterns of these tumors and hence no absolute standard is accepted. S-100 protein has been shown to have a broad distribution in human tissues including the renal tubules. Lin et al. studied the expression of S-100 protein in primary and metastatic renal cell carcinoma and nonneoplastic renal tissue [13]. S-100 protein has been identified in approximately 50–90% of MPNSTs [14]. Shimada et al. have worked on new markers such as nestin in addition to S-100 protein positivity and cytokeratin negativity as a supplementary tool for confirmation of MPNST [15]. Nestin is an intermediate filament protein which is expressed in neuroectodermal stem cells [15]. Additionally, Leu-7 (CD-57) and myelin basic protein are used frequently to assess neural differentiation of a neoplasm, noted in 50% and 40% of cases, respectively [16]. In our patient positive markers including protein S-100 and CD-57 and negative ones such as cytokeratin and C-kit were consistent with the diagnosis.

The cornerstone of treatment of MPNST is complete surgical excision of the tumor to achieve wide (negative) margins. This offers the best outcome with respect to both local recurrence and distant metastases. Despite limited response, both adjuvant chemotherapy and radiation are also often employed [17, 18]. Chemotherapy can be administered in the pre- and postoperative settings. According to the literature, several cycles of adjuvant chemotherapy with cyclophosphamide/doxorubicin have been advocated [19]. Our patient received eight courses of carboplatin, doxorubicin, and ifosfamide postoperatively which showed successful results.

Ducatman et al. studied MPNST in patients with and without neurofibromatosis and reported 5-year survival rates of 16% and 53%, respectively [6]. For patients with MPNST, careful follow-up including appropriate imaging studies is required. Interestingly, our patient did not show any stigmata of von Recklinghausen neurofibromatosis clinically. Fortunately, three years after removal of the tumor our patient is well and free of tumor.

## 4. Conclusion

The case report presented an incidental occurrence of a MPNST in a child with a single kidney. Nevertheless, this anomaly cannot be generalized to every patient with MPNST. Instead, the authors suggest considering genitourinary anomalies in the setting of tumors of MPNST in children. This is also report of a case of MPNST in a boy without having any evidence of von Recklinghausen neurofibromatosis who was detected to have a retroperitoneal mass in anatomic place of right kidney who later confirmed to have unilateral renal agenesis.
